# Supplementary material for: Ultrafast imaging of spontaneous symmetry breaking in a photoionized molecular system
Source: Nat Commun. 2021 Jul 9;12:4233. doi: 10.1038/s41467-021-24309-z (PMC8270947; doi:10.1038/s41467-021-24309-z)
Supplement: Supplementary file 1 — Supplementary Information [file 41467_2021_24309_MOESM1_ESM.pdf]

Supplemental Material: Ultrafast imaging of spontaneous symmetry breaking in a photoionized molecular system

Min Li*,[1] Ming Zhang*,[2,3] Oriol Vendrell,[4] Zhenning Guo,[2] Qianru Zhu,[5] Xiang Gao,[5] Lushuai Cao,[5] Keyu Guo,[1] Qin-Qin Su,[1] Wei Cao,[1,*] Siqiang Luo,[1] Jiaqing Yan,[1] Yueming Zhou,[1] Yunquan Liu,[2,†] Zheng Li,[2,‡] and Peixiang Lu[1,6,7,§]

[1]*Wuhan National Laboratory for Optoelectronics and School of Physics, Huazhong University of Science and Technology, Wuhan 430074, China*

[2]*State Key Laboratory for Mesoscopic Physics and Collaborative Innovation Center of Quantum Matter, School of Physics, Peking University, Beijing 10087, China*

[3]*University of Science and Technology Beijing, Beijing 10087, China*

[4]*Physikalisch-Chemisches Institut, Universität Heidelberg, Im Neuenheimer Feld 229, D-69120 Heidelberg, Germany*

[5]*MOE Key Laboratory of Fundamental Physical Quantities Measurement and Hubei Key Laboratory of Gravitation and Quantum Physics, PGMF and School of Physics, Huazhong University of Science and Technology, Wuhan 430074, China*

[6]*Hubei Key Laboratory of Optical Information and Pattern Recognition, Wuhan Institute of Technology, Wuhan 430205, China*

[7]*CAS Center for Excellence in Ultra-intense Laser Science, Shanghai 201800, China*

(Dated: June 12, 2021)

PACS numbers: 32.80.Fb, 42.50.Hz, 32.80.Rm

* weicao@hust.edu.cn

† yunquan.liu@pku.edu.cn

‡ zheng.li@pku.edu.cn

§ lupeixiang@hust.edu.cn

I. COULOMB MOMENTUM MAPPING

To calculate the momentum distribution of three-body breakup channel (CH_2^++H^++H^+) from different configurations, the Coulomb explosion is simulated by the classical Hamiltonian of three point charged particles [1]

$$H = T + V_{\mathrm{Coul}}(r_1, r_2, r_C) \quad (1)$$

$$V_{\mathrm{Coul}}(r_1, r_2, r_C) = \frac{1}{r_{12}} + \frac{1}{r_{1C}} + \frac{1}{r_{2C}}\,, \quad (2)$$

where T is the kinetic energy, r_1, r_2 and r_C are coordinates of two ionized H^+ and the mass point of CH_2^+, respectively. Molecular dynamics is carried out for 2500 fs, with the initial positions set to equilibrium geometry of $C_{3\mathrm{v}}$, $C_{2\mathrm{v}}$, $D_{2\mathrm{d}}$ and initial momentum set to zero.

The configuration evolution of CH_4^+ can be revealed by momentum distribution of CH_2^+-H^+-H^+ three-body explosion result. Each event can be classified into one of these symmetry configurations according to the distance in momentum space. The i-th event is described by the four coordinates in Newton plot $p^{(i)}_{x,\mathrm{CH}_2^+}, p^{(i)}_{y,\mathrm{CH}_2^+}, p^{(i)}_{x,\mathrm{H}^+}, p^{(i)}_{y,\mathrm{H}^+}$ and the momentum of another H^+ ion is set to be $(1, 0)$. The distance between the i-th event and one of the $C_{3\mathrm{v}}$ configurations is defined by

$$\begin{aligned} d^2_{i,C_{3\mathrm{v}}} = & \ (p^{(i)}_{x,\mathrm{CH}_2^+} - p^{(C_{3\mathrm{v}})}_{x,\mathrm{CH}_2^+})^2 + (p^{(i)}_{y,\mathrm{CH}_2^+} - p^{(C_{3\mathrm{v}})}_{y,\mathrm{CH}_2^+})^2 \\ & + (p^{(i)}_{x,\mathrm{H}^+} - p^{(C_{3\mathrm{v}})}_{x,\mathrm{H}^+})^2 + (p^{(i)}_{y,\mathrm{H}^+} - p^{(C_{3\mathrm{v}})}_{y,\mathrm{H}^+})^2 \,. \end{aligned} \quad (3)$$

The distance between event i and $C_{3\mathrm{v}}$ is defined by the minimum value of its distance to each different $C_{3\mathrm{v}}$ configuration and so do $C_{2\mathrm{v}}$ and $D_{2\mathrm{d}}$. Each exploded CH_4^+ is classified to the symmetry of minimal distance. The configuration evolution of CH_4^+ and comparison with MD simulation of two-body explosion channels is shown in Fig. S1.

II. MOLECULAR DYNAMICS SIMULATION DETAILS

MCSCF method and DZV basis are used for CH_4^+ configuration evolution including an active space of 7 orbitals. Only $1s$ orbital of C atom is regarded as core orbital and doubly occupied. For each time point in the CH_4^+ trajectory, dication occurs at the instant of the probe pulse. MCSCF method and DZV basis are used for the propagation of CH_4^{2+} trajetories.

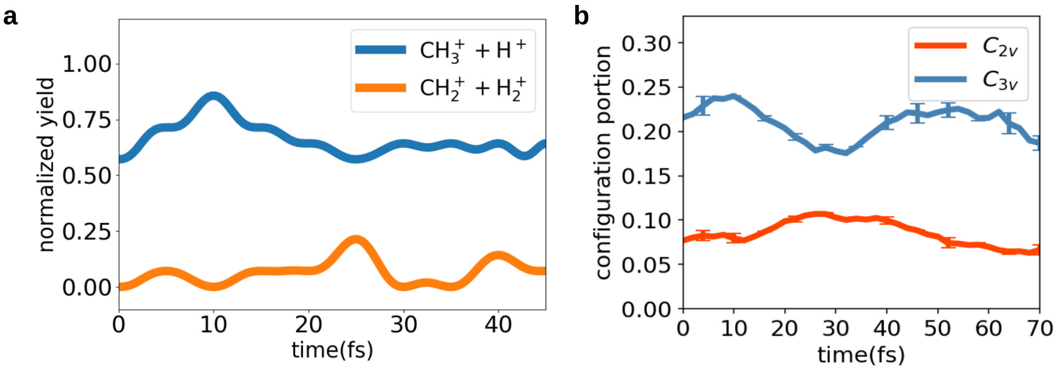

FIG. S1. **Temporal evolution in symmetry space.** Comparison between **a** simulated two-body explosion result and **b** configuration evolution of CH_4^+ from three-body measurement data. The error bars in **b** represent the mean absolute deviation of the statistical errors. The 15 fs time difference from $CH_3^+ + H^+$ channel to $CH_2^+ + H_2^+$ channel is consistent with the 20 fs experimental time delay, which corresponds to the 20 fs time difference between $C_{3\mathrm{v}}$ peak and $C_{2\mathrm{v}}$ peak.

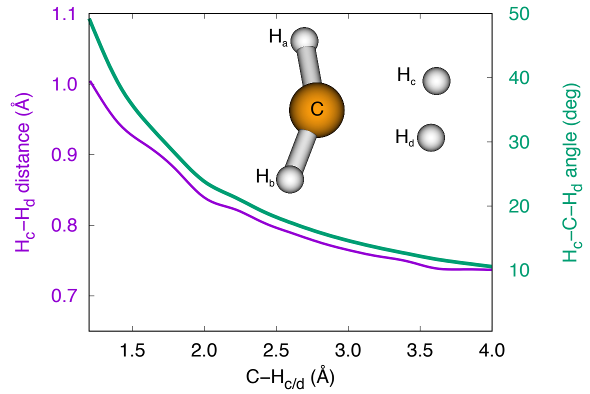

FIG. S2. **Breakup path optimized for the ground state ($^1A_1$) of CH_4^{2+} dication.** The minimal energy geometric parameters for the methane cation with stretched C-$H_{c/d}$ bonds. The purple line is for the H_c-H_d distance and the thick green line is for the H_c-C-H_d angle.

To demonstrate the fragmentation channel from the $C_{2\mathrm{v}}$ geometry of methane cation to $CH_2^+ + H_2^+$, we calculate the pathway of the Coulomb explosion after double ionization in the $^1A_1$ ground state of $C_{2\mathrm{v}}$ symmetry. The structures along the path were determined for each given C-H_c and C-H_d distances of the leaving hydrogen atoms. As shown in Fig. S2 , the H_c-H_d distance stays short and the H_c-C-H_d angle stays small in the lowest $^1A_1$ state for a very long C-H bond, which indicates the $CH_2^+ + H_2^+$ fragmentation for the $C_{2\mathrm{v}}$ geometry. Besides, a schematic dephasing and revival process of three vibrational modes leading to $C_{2\mathrm{v}}$ geometry

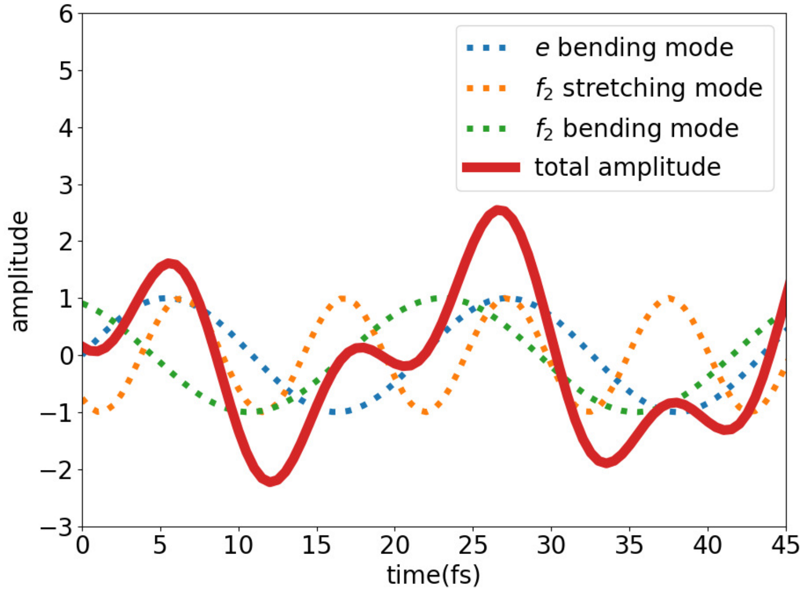

FIG. S3. **Multi-mode dynamics of Jahn-Teller distortion.** Revival and dephasing processes of e bending mode, f_2 stretching mode and f_2 bending mode of equal amplitude. The peaks at ∼5 fs and ∼25 fs are in good agreement with $CH_2^+ + H_2^+$ channel of Fig. 4a in the main text. The harmonic frequencies and corresponding vibrational periods are adapted from Ref. [2], $\nu(a_1) = 3077\text{cm}^{-1}$ (10.8fs), $\nu(e) = 1534\text{cm}^{-1}$ (21.0fs), $\nu(f_2) = 3212\text{cm}^{-1}$ (10.4fs), and $\nu'(f_2) = 1349\text{cm}^{-1}$ (24.7fs), where the a_1 mode cannot change symmetry and hence does not participate in the JT distortion.

is shown in Fig. S3. The total amplitude fluctuates due to their commensurate frequencies. The peaks of total vibration means CH_4^+ approaches C_{2v} configuration, which indicates more contribution to $CH_2^+ + H_2^+$ channel.

The initial condition of the classical MD simulation is taken for the geometries in the vicinity of the T_d symmetric configurations, and thermal velocities at experimental temperature. It means that to a good approximation mostly the DOFs of ground vibrational state of the system are populated, and is a good approximation of the quantum mechanical distribution sampling, e.g. Wigner sampling, in which for each normal mode the trajectories are set on the corresponding Bohr orbit. The error originated from variation of initial velocity should be minor, because the thermal energy of CH_4 molecule at 300K is ∼0.1eV, it is order of magnitude smaller than the stabilization energy of 1.5eV, which dominates dynamical JT process.

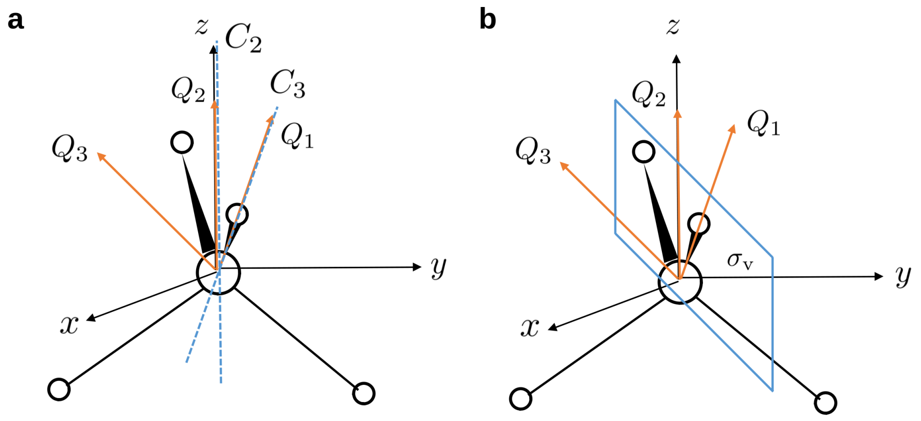

FIG. S4. **Symmetry elements conserved under Q_1, Q_2, Q_3 distortion.** **a** The distortion Q_1 is invariant under symmetry operation C_3, and Q_2 is invariant under C_2. **b** The distortions Q_1, Q_2, Q_3 remain invariant under symmetry operation $\sigma_{\rm v}$.

III. THE CORRESPONDENCE OF VIBRATIONAL MODES AND SYMMETRY DISTORTIONS

Here we analyse the distorted geometry of Jahn-Teller molecules under specified vibrational coordinates [3, 4]. Although the molecule under JT effect is forced to distort, the nuclear configuration with maximum symmetry must be preferentially produced.

For the methane cation of T_d point group, from the character table, one typical basis of f_2 vibration is (x, y, z). The space spanned by basis (x, y, z) is called distortion space, because a set of f_2 vibrational coordinates have identical transformation properties to f_2 representation basis (x, y, z). For any element in the distortion space, there are some conserved symmetry operations, which correspond to the operations of the same character to the identity operator. These operations form a group denoted $K(T_d, f_2)$. From the character table

$$K(T_d, f_2) = C_1 \,. \tag{4}$$

If there exists some special distortions, such that more symmetry operations of the original point group are conserved, these symmetry operations form a group belonging to $E(T_d, f_2)$, which is a set of subgroups of T_d.

A symmetry element will remain symmetric under a distortion if and only if this symmetry operation leaves the distortion invariant. For example, under distortion $Q_1 = x + y + z$, some symmetry operations (besides identity element E) will conserve, such as C_3 and $\sigma_{\rm v}$ in

Fig. S4

$$C_3 Q_1 = C_3 x + C_3 y + C_3 z = y + z + x = Q_1 \tag{5}$$

$$\sigma_{\rm v} Q_1 = \sigma_{\rm v} x + \sigma_{\rm v} y + \sigma_{\rm v} z = y + x + z = Q_1 \, . \tag{6}$$

All these symmetry operations form a group $C_{3\rm v}$. Similarly, under distortion $Q_2 = z$, symmetry operations such as C_2 and $\sigma_{\rm v}$ will conserve

$$C_2 Q_2 = C_2 z = z = Q_2 \tag{7}$$

$$\sigma_{\rm v} Q_2 = \sigma_{\rm v} z = z = Q_2 \, , \tag{8}$$

they form a group $C_{2\rm v}$. And under $Q_3 = -(x + y)$, only $\sigma_{\rm v}$ will conserve

$$\sigma_{\rm v} Q_3 = \sigma_{\rm v}(-x) + \sigma_{\rm v}(-y) = -(y + x) = Q_3 \, , \tag{9}$$

which corresponds to point group C_s. In conclusion,

$$E(T_d, f_2) = \{C_{3\rm v}, C_{2\rm v}, C_s\} \, . \tag{10}$$

So there exist some special distortions, such that the distorted geometry can remain in higher symmetry. Thus we know that f_2 mode leads to $C_{3\rm v}$ and $C_{2\rm v}$ distorted geometry and the maximal symmetric $C_{3\rm v}$ is preferred.

Similarly, for e representation

$$K(T_d, e) \; = \; D_2 \tag{11}$$

$$E(T_d, e) \; = \; D_{2\rm d} \, . \tag{12}$$

A typical basis for e representation of T_d point group is $(2z^2 - x^2 - y^2, x^2 - y^2)$. Obviously, there exists a distortion $Q_4 = 2z^2 - x^2 - y^2$ which remains unchanged under the symmetry operations in $D_{2\rm d}$ subgroup.

Hence, CH_4^+ is distorted to $D_{2\rm d}$ geometry due to e bending mode, and f_2 mode leads to $C_{3\rm v}$ and $C_{2\rm v}$ distorted geometry, as shown in Fig.4 in the main text. See Ref.[3] for the expression for each vibrational coordinate. Both f_2 mode and e mode contribute to $CH_2^+ + H_2^+$ channel, whose dephasing and revival process correspond to the second peak of simulation results as well as ∼20fs delay from experiment. However, only the f_2 stretching mode will lead to $CH_3^+ + H^+$ channel.

IV. WAVE PACKET DYNAMICS SIMULATION

To understand the dynamics of the JT distortion, we carried out wave packet dynamics simulation using the multi-configuration time-dependent Hartree (MCTDH) method [5], based on an effective Hamiltonian by considering molecular symmetry. In the Hamiltonian, the potential of the corresponding $F \otimes (f \oplus e)$ JT effect of CH_4^+ is mapped onto the surface of a sphere [6, 7], where the e and f_2 vibrational modes are parametrized by spherical harmonics of order $l = 2$ (Atomic units (a.u.) are used throughout unless specified otherwise),

$$\begin{aligned} Q_{\mathrm{e}'} &= d\left[3(\cos\theta)^2 - 1\right] \\ Q_{\mathrm{e}''} &= d\sqrt{3}(\sin\theta)^2\cos(2\phi) \\ Q_{\mathrm{f}'} &= d\sqrt{3}\sin(2\theta)\sin\phi \\ Q_{\mathrm{f}'} &= d\sqrt{3}\sin(2\theta)\cos\phi \\ Q_{\mathrm{f}'''} &= d\sqrt{3}(\sin\theta)^2\sin(2\phi)\,, \end{aligned} \tag{13}$$

where θ and ϕ are the parameterized coordinates and $d = 0.373$ Å is a parametrized distortion parameter [7].

The linear JT effect of f_2 vibration produces methane cation in $C_{3\mathrm{v}}$, which contributes to the fragmatation into $CH_3^+ + H^+$. Because of the degenerate ground state, this structure undergoes a JT effect to $C_{2\mathrm{v}}$ geometry[8], which dissociates to $CH_2^+ + H_2^+$. The JT deformation dynamics is governed by the parametric model Hamiltonian [7],

$$\begin{aligned} \hat{H} &= \hat{T} + \hat{V}(\theta,\phi) \\ \hat{T} &= -t\left[\tfrac{1}{\sin\theta}\tfrac{\partial}{\partial\theta}\left(\sin\theta\tfrac{\partial}{\partial\theta}\right) + \tfrac{1}{\sin^2\theta}\tfrac{\partial^2}{\partial\phi^2}\right] \\ \hat{V}(\theta,\phi) &= V_0 + \sqrt{\tfrac{7}{12}}\left[Y_{4,0} + \sqrt{\tfrac{5}{14}}\left(Y_{4,4} + Y_{4,-4}\right)\right]V_4 \\ &\quad + \tfrac{1}{\sqrt{8}}\left[Y_{6,0} - \sqrt{\tfrac{7}{2}}\left(Y_{6,4} + Y_{6,-4}\right)\right]V_6\,, \end{aligned} \tag{14}$$

where $t = 10$ cm^{-1} is the pseudo-rotation parameter, the potential parameters are chosen to be $V_0 = 0$, $V_4 = -2260$ cm^{-1}, $V_6 = 3100$ cm^{-1}, which approximately reproduce the JT stablization energy and the *ab initio* energy values of the methane cation [7]. The initial wavepacket is prepared in vicinity of the symmetric points on the surface of the sphere, which represents the $C_{3\mathrm{v}}$ configurations with a contracted C-H bond, and favors

the $CH_3^+ + H^+$ breakup channel in the laser-induced Coulomb explosion. Following the Franck-Condon principle, the FWHM width of the initial wavepacket $\Psi(\theta, \phi)$ is set such that the corresponding E vibrational coordinates $Q_{e'}$ and $Q_{e''}$ have the width of the ground state wave function [7]. To analyse the population of C_{2v} configurations in the final state wavepacket, the wavepacket densities in the vicinity $(\Delta\theta, \Delta\phi)$ of the C_{2v} points on the sphere are integrated, where $(\Delta\theta, \Delta\phi)$ is phenomenologically taken as the width of the initial wavepacket. The potential energy surface and the time dependence of the population is shown in Figs. 5a and 5b of the main text. One can see that the population in the D_{2d} configuration is much smaller than that in the C_{2v} configuration. Moreover, the population of the C_{2v} configurations peaks at ∼18.7 fs, which is almost consistent with the measurement. Using this time delay between the two breakup channels, we can reproduce the experimental data shown in Fig. 2 of the main text. The KER of the fragments from the Coulomb explosion is related to the distance R between the centers of mass for the $CH_2^+ + H_2^+$ and $CH_3^+ + H^+$ fragments at the moment of separation by $\text{KER} \approx 1/R$. We assume that the reduced mass of the nuclei starts moving with constant velocity, thus the distance R can be expressed as $R \approx R_0 + v_0 t$, where R_0 is the initial distance and v_0 is the velocity adjusted to best fit with the experiment. With a time delay of 18.7 fs between the two breakup channels, we achieve a good agreement between the simulation and the measurement, as shown in Fig. 2 of the main text.

V. LASER POWER DEPENDENCY CHECK OF THE THREE-BODY BREAKUP CHANNELS

Under condition that the pump and probe pulses are of identical wavelength and intensity, it could thus well be that the results of the three-body breakup contain a superposition of two pathways, (i) pump from neutral to cation, probe from cation to dication, and (ii) pump from neutral to dication, probe from dication to trication.

In order to clarify that the three-body breakup yields are dominantly contributed by the cation before the probe pulse, we have carried out the laser power dependency check experimentally for the three-body Coulomb explosion channel by reducing the intensity of pump pulse to 1/4 ($\sim 0.7\times10^{14}\text{W/cm}^2$) and keeping the intensity of probe pulse invariant ($\sim 3\times10^{14}\text{W/cm}^2$). Under this condition, the probability of double ionization by the pump

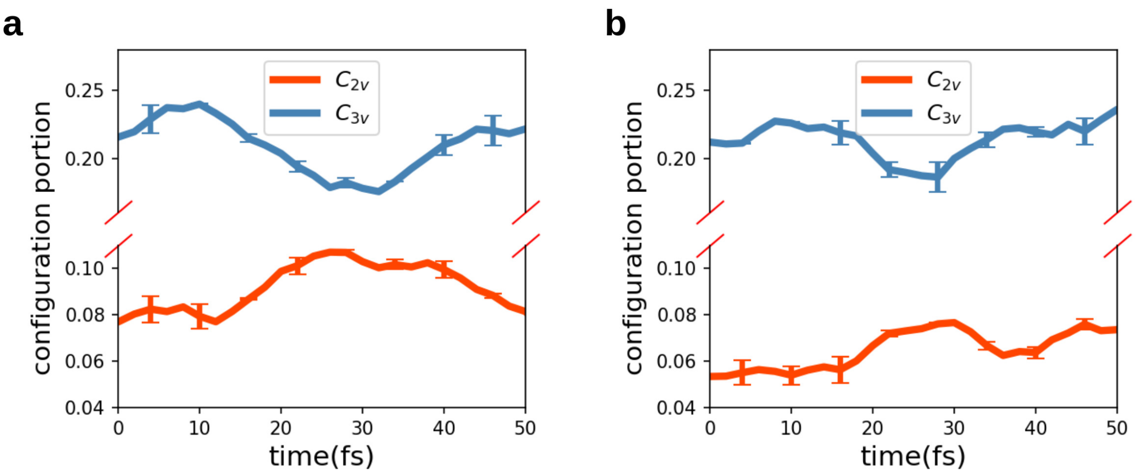

FIG. S5. **Three-body Coulomb explosion probe at various pump intensities.** Temporal evolution of $C_{3\mathrm{v}}$ like and $C_{2\mathrm{v}}$ like symmetry configurations of **a** $\sim 3 \times 10^{14}\mathrm{W/cm^2}$ pump pulse intensity and **b** reduced pump pulse of intensity $\sim 0.7 \times 10^{14}\mathrm{W/cm^2}$ with mean absolute error, the delays between $C_{3\mathrm{v}}$ to $C_{2\mathrm{v}}$ peaks are 20 fs ($3 \times 10^{14}\mathrm{W/cm^2}$ pump) and 22 fs ($0.7 \times 10^{14}\mathrm{W/cm^2}$ pump), respectively. The configuration evolution from $C_{3\mathrm{v}}$ to $C_{2\mathrm{v}}$ as well as the delay between these two peaks are both insensitive to the change of pump pulse intensity.

pulse must be substantially reduced. As a result, the relative portion of dication after the pump pulse should become smaller, i.e., the pathway (ii) is suppressed under this condition. We present the result of the temporal evolution for configurations of specific symmetries $C_{3\mathrm{v}}$ and $C_{2\mathrm{v}}$ under reduced pump pulse intensity. The qualitative consistency of the power dependency check reflects the fact that the pathway (ii) can be removed when understanding the temporal evolution of configurations shown in the Fig. S5. We further measured the mass to charge ratio (m/q) spectra of ionic species for the pump laser pulse alone with various intensities, from which the yields of $\mathrm{CH_4^{2+}}$ and $\mathrm{CH_4^+}$ can be directly determined. Because part of the $\mathrm{CH_4^{2+}}$ dication could dissociate to $\mathrm{CH_3^+}$+$\mathrm{H^+}$ and $\mathrm{CH_2^+}$+$\mathrm{H_2^+}$, we extract the number of the two dissociation events in the photoion-photoion coincidence spectra (PIPICO) for various pump intensities. Subtracting the number of $\mathrm{CH_3^+}$+$\mathrm{H^+}$ events from the counts of $\mathrm{CH_3^+}$ in the m/q spectra gives us the yield of the $\mathrm{CH_3^+}$+$\mathrm{H^+}$ channel. The final results of dication to cation ratio analysis are presented in Fig. S6. Even at highest pump intensity of $3 \times 10^{14}\mathrm{W/cm^2}$, the ratio of dication (sum yield of three channels of $\mathrm{CH_4^{2+}}$, $\mathrm{CH_3^+}$+$\mathrm{H^+}$, and $\mathrm{CH_2^+}$+$\mathrm{H_2^+}$) to cation (sum yield of two channels of $\mathrm{CH_4^+}$ and $\mathrm{CH_3^+}$+$\mathrm{H^+}$) is $\sim 3.5 \times 10^{-2}$,

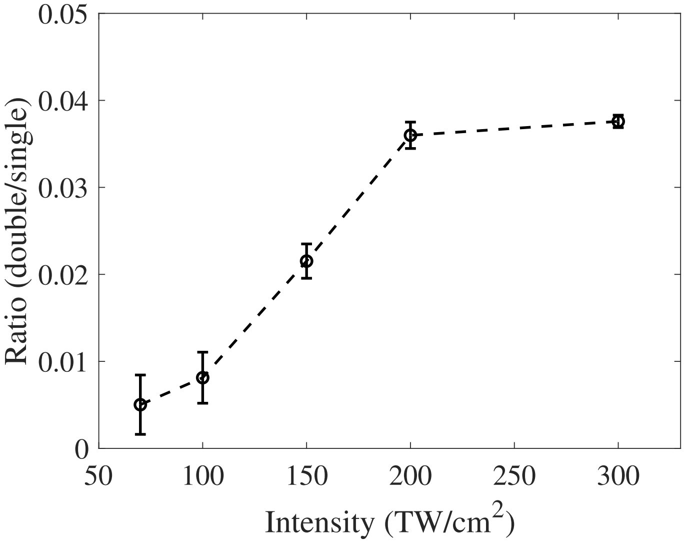

FIG. S6. **The ratio of dication to cation of methane molecules at various pump intensities**. The ratios are determined from mass to charge spectra and photoion-photoion coincidence spectra (PIPICO). The error bars show the standard deviation of the statistical errors.

and is of 5×10^{-3} at lower pump intensities used for the power dependency check. The signals at the mass to charge ratios for CH^{2+} and CH^{3+} species do not exhibit peaks above background level. The small ratio of dication species could be resulted from its relatively high double ionization potentials, the lowest of which is calculated to be 23.43 eV on the SA-CASSCF(7,7)//aug-cc-pVTZ (cation) and SA-CASSCF(6,7)//aug-cc-pVTZ (dication) level by the complete active space self-consistent field method. It explains the negligible contribution of dication to the JT dynamics of methane cation initiated by the pump pulses of $0.7 - 3 \times 10^{14}$W/cm^2 intensity.

---

[1] Li, Z. et al. Ultrafast isomerization in acetylene dication after carbon K-shell ionization. *Nature Comm.* **8**, 453 (2017).

[2] Mondal, T. & Varandas, A. J. C. Structural evolution of the methane cation in subfemtosecond photodynamics. *J. Chem. Phys.* **143**, 014304 (2015).

[3] B.Bersuker, I. *The Jahn-Teller Effect.* (Cambridge University Press, 2006).

[4] Ceulemans, A. & Vanquickenborne, L. G. The epikernel principle. *Structure and Bonding* **66**,

125–158 (1989).

[5] Beck, M. H., Jäckle, A., Worth, G. A. & Meyer, H.-D. The multiconfiguration time-dependent Hartree method: A highly efficient algorithm for propagating wavepackets. *Phys. Rep.* **324**, 1–105 (2000).

[6] Chancey, C., O'Brien, M. & O'Brien, M. *The Jahn-Teller effect in* C_{60} *and other icosahedral complexes.* (Princeton University Press, 1997).

[7] Jacovella, U., Wörner, H. J. & Merkt, F. Jahn-teller effect and large-amplitude motion in CH_4^+ studied by high-resolution photoelectron spectroscopy of CH_4. *J. Mol. Struct.* **343**, 62–75 (2018).

[8] Frey, R. F. & Davidson, E. R. Potential energy surfaces of CH_4^+. *J. Chem. Phys.* **88**, 1775–1785 (1988).
